# Infant-Feeding in Its Relation to Health and Disease

**Published:** 1902-02

**Authors:** 


Infant-Feeding in its Relation to Health and Disease. By Louis Fischer, M. D., Visiting Physician to the Willard Parker and Reception Hospitals of New York City, etc. Duodecimo, pp. 351. 52 illustrations, with 23 charts and tables, mostly original. Second edition. Philadelphia: F. A. Davis Co. 1901.

Dr. Fischer's valuable little book on infant feeding met with such a hearty reception that a new edition was demanded in six months. This second edition appears in an entirely new form and dress, with numerous changes and additions. The alterations are all in the way of improvement.—M.J.F.

